# The Role of Polydeoxyribonucleotide as a Biotherapy for Musculoskeletal Disorders

**DOI:** 10.3390/ijms27167304

**Published:** 2026-08-16

**Authors:** Jaeseok Choi, Yeong-Min Yoo

**Affiliations:** Institute of Environmental Research, Kangwon National University, Chuncheon 24341, Republic of Korea; gobiobotia@kangwon.ac.kr

**Keywords:** polydeoxyribonucleotide, adenosine A2A receptor, tendinopathies, musculoskeletal disorders, spinal radiculopathy, orthopedic rehabilitation

## Abstract

Polydeoxyribonucleotide (PDRN) is a DNA-derived biological therapy gaining prominence in the treatment of musculoskeletal disorders. It functions as an adenosine A2A receptor (A2AR) agonist and serves as a substrate in the nucleotide salvage pathway. These mechanisms drive anti-inflammatory effects, stimulate angiogenesis via vascular endothelial growth factor, and promote collagen synthesis. In osteoarthritis, PDRN reduces cartilage degradation and promotes the chondrogenic differentiation of stem cells. PDRN has shown therapeutic potential in tendinopathies, including Achilles and rotator cuff injuries, by supporting tissue repair. Preliminary reports suggest it may offer a non-steroidal alternative for managing spinal radiculopathy when corticosteroids are contraindicated, though robust comparative trials are needed. Preclinical and clinical evidence indicate favorable preliminary safety profiles and potential pain reduction. Future large-scale trials are needed to standardize the dosing protocols for integrated orthopedic rehabilitation.

## 1. Introduction

Polydeoxyribonucleotide (PDRN) is an emerging regenerative agent with well-characterized anti-inflammatory, tissue-repairing, and angiogenic properties that has attracted increasing attention in orthopedics and musculoskeletal medicine [1]. This literature review synthesizes the current evidence from in vitro, in vivo, preclinical, and clinical studies on the mechanisms of action and therapeutic applications of PDRN across a spectrum of musculoskeletal conditions, including osteoarthritis, tendinopathies, bone regeneration, ligament injuries, and spinal disorders.

### 1.1. Biochemical Composition and Source

PDRN is a proprietary registered drug classified as a mixture of deoxyribonucleotide polymers with molecular weights ranging from 50 to 1500 kDa, with the most abundant molecular weight peaking at approximately 132 kDa [2,3,4,5]. Originally approved by the Italian Medicines Agency (AIFA) in 1994 for tissue repair, superficial wounds, and dystrophic connective tissue disorders [2,6], PDRN has since been utilized as a registered prescription agent across several jurisdictions, particularly in South Korea and Italy, for wound healing, tissue regeneration, and various musculoskeletal indications [2,6]. It is derived from the controlled purification and sterilization of sperm DNA from *Oncorhynchus mykiss* (Salmon Trout) or *Oncorhynchus keta* (Chum Salmon), a procedure that ensures the absence of active proteins and peptides that may trigger immune reactions [2,3,4,5]. Sperm cells are considered the most appropriate source of raw materials because they provide highly purified DNA without the risk of contamination by peptides, proteins, and lipids that may remain as impurities when somatic cells are used [2,3,4,5]. The resulting product consists of approximately 50% double-stranded deoxyribonucleotides, distinguishing it from related drugs such as defibrotide, which is derived from porcine intestinal mucosa DNA and possesses a significantly lower molecular weight of 16–20 kDa [2]. The pharmacokinetics of PDRN reveal a bioavailability of approximately 80–90% after intramuscular administration, with peak plasma levels at approximately 1 h post-injection and a half-life of approximately 3–3.5 h. The drug stimulates a cascade of downstream events that extend far beyond its plasma half-life [2,6]. PDRN is not metabolized by the liver; instead, it is degraded by nonspecific plasma DNA nucleases into oligonucleotides and mononucleotides and is excreted primarily in the urine (~65%) and, to a lesser extent, in feces [2].

### 1.2. Mechanisms of Action of PDRN

The most established mechanism of action of PDRN is the activation of the adenosine A2A receptor (A2AR), a G protein-coupled receptor (GPCR) involved in regulating inflammation, oxygen consumption, ischemia, cell proliferation, and angiogenesis [7]. Adenosine signals through four receptor subtypes, and PDRN appears to act preferentially on A2AR, as shown by the loss of its biological effects after treatment with the A2AR antagonist 3,7-dimethyl-1-propargylxanthine (DMPX) in fibroblast cultures and animal models [2]. Through A2AR activation, PDRN increases the expression of vascular endothelial growth factor (VEGF), promotes cell differentiation, supports fibroblast maturation and collagen synthesis, and accelerates granulation tissue formation and repair [7]. This pathway also mediates anti-inflammatory effects by suppressing tumor necrosis factor alpha (TNF-α), interleukin-6 (IL-6), and interleukin-1beta (IL-1β), and increasing IL-10 [2,8,9]. A2AR signaling activates adenylyl cyclase, increases cyclic adenosine monophosphate (cAMP), and subsequently stimulates protein kinase A (PKA) and downstream transcription factors, including nuclear factor kappa B (NF-κB) and cAMP-response element-binding protein (CREB) [9].

In addition to receptor-mediated signaling, PDRN acts via the salvage pathway, in which nucleosides and nucleotides generated by PDRN degradation support DNA synthesis in damaged or ischemic tissues with a limited capacity for de novo synthesis, thereby restoring normal cell proliferation and growth [2,10,11]. This combination of A2AR agonism and salvage pathway support confers PDRN with tissue-repairing properties that distinguish it from other DNA-derived products with different molecular weights, sources, or manufacturing processes [2].

PDRN also promotes angiogenesis and extracellular matrix (ECM) remodeling [7]. It enhances angiogenesis by stimulating VEGF and other key growth factors [7]. Improved vascularization supports oxygen and nutrient delivery to injured tissues and facilitates the removal of metabolic waste, both of which are essential for effective healing [7]. In addition, PDRN promotes collagen synthesis and modulates matrix metalloproteinase-1 (MMP-1) activity, thereby supporting tissue remodeling and improving the structural integrity and mechanical strength of healing musculoskeletal tissue [7]. Preclinical studies have shown that PDRN enhances tendon repair by increasing collagen synthesis [12].

In specific musculoskeletal tissues, these mechanisms are expressed as follows:

▪ Tendon healing: PDRN enhances tendon repair by suppressing inflammation and apoptosis while promoting collagen synthesis [11]. Animal models of rotator cuff repair have shown improved healing and reduced fatty degeneration following PDRN treatment [12].
▪ Bone regeneration: PDRN stimulates osteoblast activity and angiogenesis, which are essential for bone healing and regeneration. In an in vivo experimental study, PDRN promoted early bone formation during lateral bone augmentation with immediate implant placement [13,14].
▪ Cartilage protection and regeneration: PDRN may reduce cartilage degradation and promote chondrogenic differentiation [15]. In human bone marrow-derived mesenchymal stem cells (hBMSCs), PDRN alleviated IL-1β-induced impairment of chondrogenic differentiation, suggesting a role in cartilage repair in osteoarthritis [15,16]. Canine osteoarthritis cell models have also shown anti-inflammatory effects, including reduced inflammation and apoptosis following PDRN treatment [17].
▪ Anti-inflammatory and anti-apoptotic effects: PDRN consistently exerts anti-inflammatory and anti-apoptotic effects across musculoskeletal tissues [7,12,15,17]. Its anti-apoptotic activity, including a reduced Bax/Bcl-2 ratio and decreased activation of caspase-3 and caspase-9, further supports tissue protection following mechanical or inflammatory injury [18]. This helps to limit cellular damage and creates a more favorable environment for healing [7].

Overall, the effects of PDRN on musculoskeletal tissues are driven by modulation of inflammation, promotion of cell proliferation and differentiation, stimulation of angiogenesis, and support of ECM remodeling, primarily through A2AR activation and nucleotide provision [7].

As a narrative review, this manuscript synthesizes findings across a spectrum of evidence levels, ranging from mechanistic in vitro experiments and animal models to clinical trials and case series. While preclinical studies elucidate molecular pathways, their findings cannot be directly extrapolated to clinical outcomes without validation in rigorous clinical trials.

## 2. The Effects of PDRN Treatment or Therapy in Osteoarthritis and Cartilage Repair

### 2.1. The Mechanisms of PDRN in Osteoarthritis and Cartilage Repair

PDRN treatment has shown therapeutic potential in osteoarthritis and cartilage repair through anti-inflammatory effects, promotion of chondrogenic differentiation, and inhibition of cartilage degradation [15,16]. These effects are primarily mediated by the activation of A2ARs and provision of essential nucleotides through the salvage pathway [15,16].

Inflammation modulation is the key mechanism underlying the action of PDRN in osteoarthritis. Osteoarthritis is characterized by chronic inflammation, particularly elevated levels of pro-inflammatory cytokines such as IL-1β, which contribute to cartilage degradation [15,16]. As an A2AR agonist, PDRN inhibits pro-inflammatory signaling pathways, including NF-κB, thereby reducing the production of inflammatory mediators and attenuating the inflammatory response that exacerbates osteoarthritis pathology [15,16]. In hBMSCs, PDRN reduced chondrogenic differentiation in response to IL-1β-induced impairment, indicating a protective effect on cartilage-forming cells [15]. In a canine osteoarthritis cell model, PDRN exhibited anti-inflammatory activity, further supporting its potential as a treatment for osteoarthritis [15,16].

PDRN promotes tissue regeneration and cartilage repair by supplying purine and pyrimidine bases, deoxyribonucleosides, and deoxyribonucleotides [7,12,15]. These components serve as substrates in the salvage pathway, enabling nucleic acid synthesis. This function is particularly relevant in damaged or ischemic tissues, including osteoarthritis-affected cartilage, where de novo nucleotide synthesis may be impaired [15]. By supporting nucleic acid synthesis, PDRN promotes cell proliferation and differentiation, which are essential for cartilage repair and regeneration [15,16]. Its ability to promote the proliferation of hBMSCs further substantiates its direct involvement in cartilage formation [15,16]. Chondrogenic differentiation is the process by which mesenchymal stem cells differentiate into chondrocytes, the cells responsible for producing and maintaining the cartilage matrix.

In addition, PDRN contributes to ECM remodeling, which is essential for functional cartilage repair. It promotes collagen synthesis, a major structural component of cartilage [15]. Proper collagen production and organization are necessary to restore the mechanical strength and integrity of damaged cartilage. By modulating ECM remodeling, PDRN may promote the formation of more organized functional cartilage than inferior scar tissue [15].

Overall, PDRN appears to act through coordinated anti-inflammatory, A2AR-mediated, and tissue regenerative mechanisms, including nucleotide provision and chondrogenic differentiation. Together, these effects may reduce cartilage degradation, support tissue repair, and improve functional recovery in osteoarthritis [15]. Although the precise numerical outcomes require a review of the original figures, preclinical evidence suggests that PDRN is a promising therapeutic approach for osteoarthritis [15].

### 2.2. Preclinical and Clinical Studies in Osteoarthritis and Cartilage Repair

Knee osteoarthritis and chondropathy are among the most common degenerative cartilage disorders in older adults, and the limited regenerative capacity of articular cartilage makes treatment particularly challenging [9]. In preclinical in vitro studies, PDRN promotes physiological ECM accumulation in primary chondrocytes while reducing MMP-2 and MMP-9 activity, thereby decreasing proteoglycan degradation [7,19,20,21]. PDRN also acts synergistically with glucosamine to reduce ECM gene expression, further limiting cartilage matrix degradation [2,20]. As a polynucleotide, PDRN has viscoelastic properties because of its ability to bind large amounts of water, which may help reorganize the cartilage structure and increase surface hydration after intra-articular injection [9].

In a collagen-induced arthritis mouse model, PDRN significantly improved clinical signs of arthritis, reduced histological damage, lowered both cartilage expression and circulating levels of HMGB-1, TNF-α, and IL-6, and markedly increased IL-10 expression; these effects were abolished by co-administration of the A2AR antagonist DMPX [8]. In hBMSCs exposed to IL-1β-induced inflammatory stimuli, PDRN enhanced chondrogenic differentiation, increased safranin O and alcian blue staining, upregulated chondrogenic gene expression, suppressed inflammatory cytokines, and reduced apoptosis through the A2AR/cAMP/PKA/CREB pathway [15].

In clinical practice, several randomized controlled trials have evaluated intra-articular PDRN injections versus hyaluronic acid (HA) in patients with knee osteoarthritis. A systematic review and meta-analysis of five randomized controlled trials found that the PDRN group achieved greater pain relief than the HA group 1 and 2 months after injection, although no significant difference was observed at 4 months [22]. Adverse events did not differ significantly between the two groups, supporting the favorable safety profile of intra-articular PDRN injection [22]. A combination of PDRN and HA (PDRN-HA) has also been proposed to stimulate greater ECM production and synoviocyte growth than HA or PDRN alone by combining the trophic effects of PDRN with the viscoelastic properties of HA [1,9]. In patients treated with PDRN-HA, synovial fluid levels of MMPs-1 and -3, IL-6, TNF-α, and prostaglandin E2 were significantly reduced compared with HA alone, which reduced only IL-6, IL-8, and PGE2 [9]. A recent concept review highlighted emerging adenosine signaling-based approaches, such as intra-articular PDRN combined with pulsed electromagnetic fields (PEMF), as promising strategies for knee chondropathy [9].

### 2.3. A Comparison of the Key Characteristics Between PDRN and HA

Table 1 compares the key characteristics of PDRN and HA. PDRN promotes anti-inflammatory, angiogenic, and tissue repair by activating A2ARs [7,12,15,16,17]. The primary function of PDRN is to modulate the microenvironment, whereas HA is a viscoelastic supplement that contributes to joint lubrication and shock absorption [1,9]. Regarding the regenerative potential, PDRN provides indirect support for regeneration, whereas HA primarily alleviates the symptoms. The onset of effects occurred gradually over several weeks for all three drugs, and the duration of efficacy was similar, lasting for several months. Regarding the regulation of angiogenesis and inflammation, PDRN plays a leading role through immune reprogramming, whereas HA exerts minimal effects. In cartilage and tendon biology, PDRN supports ECM synthesis and fibroblast activation. HA is involved in surface protection [1,7,9,12,15,16,17]. All therapeutic modalities have favorable safety profiles and a low risk of tumorigenesis but entail moderate costs and regulatory complexity. The principal indications include tendinopathy, osteoarthritis, and chronic soft-tissue injuries [1,7,9,12,15,16,17].

**Figure 1:**
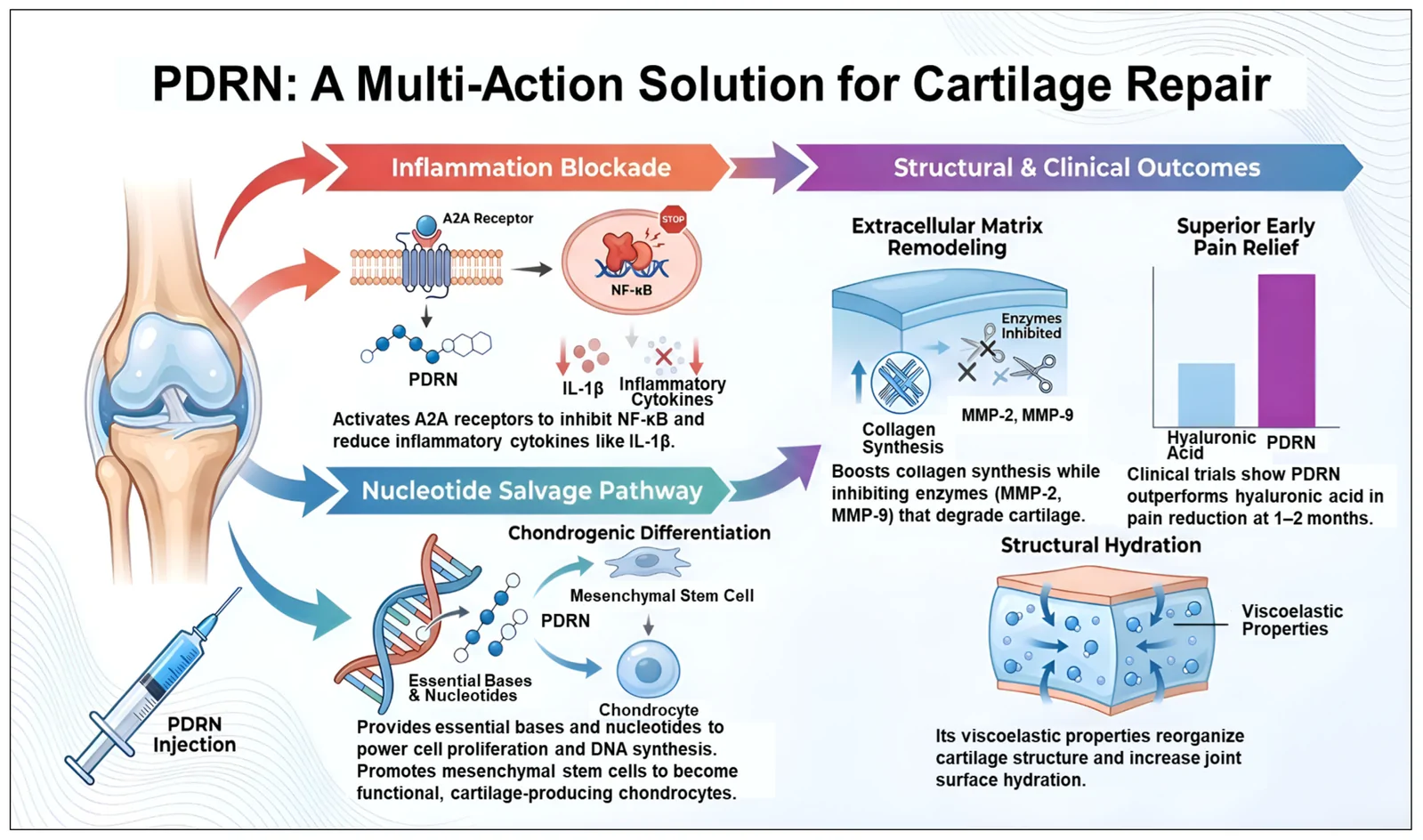
PDRN promotes cartilage repair via multiple mechanisms. Injection of PDRN activates A2ARs, inhibiting NF-κB signaling and reducing pro-inflammatory cytokines, such as IL-1β, thereby blocking inflammation. At the same time, PDRN provides the essential nucleotides and bases needed to support the proliferation and chondrogenic differentiation of mesenchymal stem cells via the nucleotide salvage pathway. Structurally, PDRN enhances ECM remodeling by promoting collagen synthesis and inhibiting matrix-degrading enzymes (MMP-2 and MMP-9). These processes result in improved cartilage hydration and viscoelastic properties, which ultimately contribute to the superior early pain relief observed in clinical outcomes compared to HA.

Figure 1 summarizes the key points: “The Effects of PDRN treatment on osteoarthritis and cartilage repair.”

## 3. The Effects of PDRN Treatment or Therapy in Tendinopathies

PDRN shows promising therapeutic effects in tendinopathies by promoting tissue regeneration, reducing inflammation, and supporting functional recovery [7,12]. Tendinopathies are common musculoskeletal disorders that account for 30–50% of sports-related injuries and are often characterized by delayed healing and incomplete restoration of structural integrity and mechanical strength [12]. PDRN may address these limitations by activating A2ARs and providing essential nucleotides via the salvage pathway [7,23].

The major mechanism of PDRN in tendinopathies involves its anti-inflammatory activity. PDRN acts as an A2AR agonist [23]. After administration, they are broken down into deoxyribonucleosides and deoxyribonucleotides, which serve as ligands for these GPCRs [23]. A2AR activation inhibits proinflammatory pathways, including NF-κB, thereby reducing the production and release of inflammatory mediators [23]. This effect helps limit tissue damage and creates a more favorable environment for tendon healing [12,23]. PDRN significantly reduces inflammation and apoptosis in rats with Achilles tendon injury [23].

In addition to modulating inflammation, PDRN supports tissue repair by supplying purine and pyrimidine bases, deoxyribonucleosides, and deoxyribonucleotides, which cells use via the salvage pathway to synthesize DNA and RNA [7,12,15]. This nucleotide supply is especially important in damaged or ischemic tendon tissues, where de novo synthesis may be impaired, because it supports cellular proliferation, differentiation, and repair. By supporting these processes, PDRN contributes to improved healing and preservation of tendon structures [7,12].

PDRN promotes angiogenesis, which is essential for tendon healing. This stimulates VEGF and other growth factors, thereby enhancing new blood vessel formation [7]. Improved vascularization supports the delivery of oxygen and nutrients to the injury site and facilitates the removal of metabolic waste products, both of which are necessary for effective tissue repair and regeneration [7].

Furthermore, PDRN contributes to ECM remodeling, which is critical for restoring the mechanical properties of injured tendons. It promotes collagen synthesis, the main structural protein in tendons [7]. Proper collagen production and organization are required to restore tendon strength and integrity, and limit scar formation, favoring more organized and functional tissues [7]. In a rat model of chronic rotator cuff tears, PDRN and polynucleotides improved tendon healing and reduced fat degeneration, suggesting a role in qualitative repair [24]. In a rabbit model of chronic rotator cuff tears, PDRN, especially when combined with microcurrent therapy, promoted the regeneration of the torn tendon [12].

Clinical evidence, including meta-analyses, supports the benefits of PDRN in patients with tendon or ligament pain [25]. Collectively, these findings suggest that PDRN is a promising treatment for tendinopathies because it supports tissue regeneration, reduces inflammation, and improves functional recovery [7,12]. Overall, both preclinical and clinical studies have indicated that PDRN may help restore tendon structure and function in tendinopathy [7,12,25].

### 3.1. Achilles Tendinopathy

In an in vivo rat Achilles tendon transection model, PDRN increased cross-sectional area and improved maximum load and tensile stress compared with controls, likely by enhancing collagen synthesis; it also increased type I collagen, FGF, VEGF, and TGF-β1, thereby improving resistance to mechanical stress [7,26]. In a separate in vivo study, PDRN reduced pain sensitivity, attenuated histological degeneration, suppressed IL-1β, IL-6, and TNF-α expression, decreased levels of cleaved caspase-3 and caspase-9, and alleviated mechanical allodynia and thermal hyperalgesia in Achilles tendon injury [7,23]. Collectively, these preclinical findings support the clinical use of PDRN in Achilles tendinopathy by demonstrating its anti-inflammatory, anti-apoptotic, and pro-regenerative effects [18].

### 3.2. Plantar Fasciitis

In a preliminary case report of chronic plantar fasciitis, 40 patients were assigned to receive either PDRN or normal saline once a week for 3 weeks. Visual analog scale (VAS) and Manchester–Oxford Foot Questionnaire scores were assessed at 4 and 12 weeks, and the PDRN group showed significantly greater improvement than the placebo group, with no reported injection-related complications [27]. In another comparative study of 44 patients with plantar fasciitis, corticosteroid injection provided faster pain relief at 2 and 6 weeks; however, no significant difference was observed at 6 months; no complications occurred in either group, suggesting that PDRN may be a long-term alternative to corticosteroids [7,28]. The proposed mechanisms underlying PDRN efficacy in plantar fasciitis include VEGF-mediated angiogenesis, increased IL-10 levels, and reduced TNF-α and IL-6 via A2AR signaling [7].

### 3.3. Rotator Cuff Tendinopathy

In a preliminary case report of 32 patients with chronic rotator cuff tendinopathy, ultrasound-guided PDRN prolotherapy significantly improved VAS, Shoulder Pain and Disability Index (SPADI), and Single Assessment Numeric Evaluation scores at 1 week, 1 month, and 3 months, with no reported complications. These findings suggest that PDRN prolotherapy is a promising non-surgical regenerative treatment option [7,29]. In a case-controlled retrospective study of 106 patients with chronic nontraumatic refractory rotator cuff disease, the PDRN injection group showed greater improvement in SPADI, VAS scores, and daily analgesic use than the control group over 3 months, although no differences were observed in isometric strength, active range of motion, or tendon tear size on ultrasonography [30]. A meta-analysis of patients with rotator cuff tendinopathy also found a significant improvement in pain after PDRN injection, although SPADI and shoulder abduction strength did not differ significantly [25]. Preclinical in vivo studies further suggested a synergistic effect of PDRN and extracorporeal shock wave therapy (ESWT) or microcurrent therapy in rabbit rotator cuff tear models, supporting the development of integrated treatment protocols [7].

### 3.4. Lateral Epicondylitis

A preliminary case report of a randomized controlled trial involving 69 patients with chronic lateral epicondylitis compared exercise alone, exercise plus a PDRN injection, and exercise plus ESWT. At 6 weeks, the PDRN group showed significantly greater improvement in the Mayo Elbow Performance Score and common extensor tendon depth on ultrasonography than the other groups, indicating a superior short-term functional benefit [7,31]. A case series reported that 2 patients with lateral epicondylitis who received ultrasound-guided PDRN injections into the common extensor tendon experienced marked pain relief within 2 weeks, with complete resolution of hypervascularity on ultrasound, consistent with PDRN’s anti-inflammatory action via reduced IL-1 and IL-6 and upregulated IL-10 [7,27,32].

### 3.5. Pes Anserine Bursitis

A preliminary case report described a 50-year-old woman with pes anserine bursitis who received an ultrasound-guided PDRN injection; the NRS score decreased from 7 to 2 at 1 week and to 0 at 2 weeks, with full range of motion maintained at 8 months and no adverse reactions [7,32,33]. The rapid and durable response aligns with PDRN’s combined anti-inflammatory and tissue-regenerative effects, particularly the reduction of pro-inflammatory cytokines and the enhancement of collagen synthesis in peritendinous structures [7].

### 3.6. Posterior Tibial Tendon Dysfunction

In a preliminary case report, a patient with posterior tibial tendon dysfunction who had persistent ankle pain despite non-steroidal anti-inflammatory drug treatment and syndesmotic surgery received PDRN prolotherapy 4 times at 1-week intervals; NRS scores decreased from 8 to 5 after the first injection and to 1 after 4 injections, with no complications reported [7,34]. The beneficial effect in this case is thought to result from PDRN-mediated VEGF stimulation, fibroblast maturation and differentiation, and reduced pro-inflammatory cytokines such as IL-6 and TNF-α [7].

**Figure 2:**
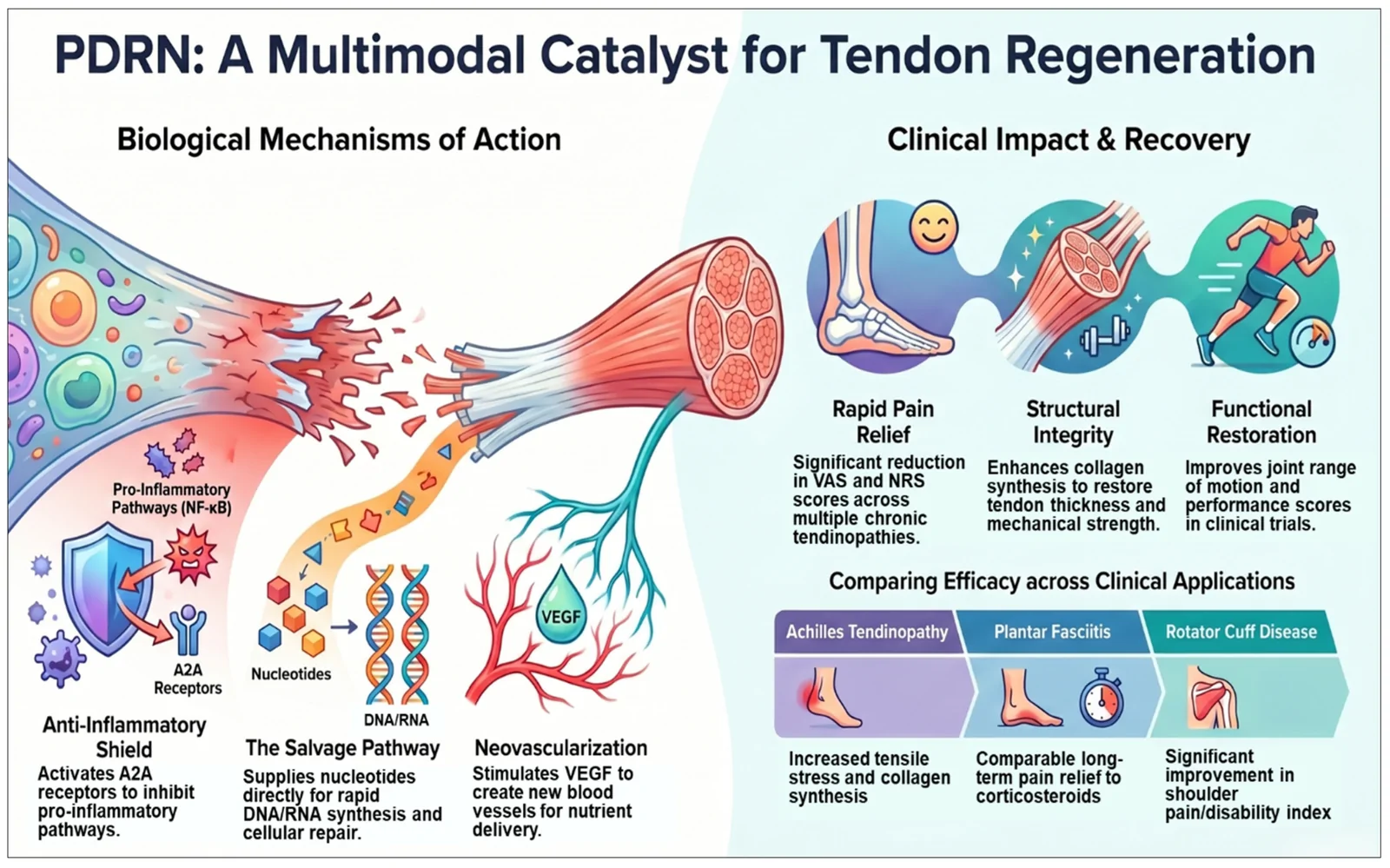
PDRN acts as a multimodal catalyst for tendon regeneration via various biological mechanisms. PDRN activates A2ARs, which inhibit pro-inflammatory pathways and provide an anti-inflammatory shield. At the same time, PDRN provides the essential nucleotides for rapid DNA/RNA synthesis, supporting cellular repair via the salvage pathway. Furthermore, PDRN stimulates VEGF-mediated neovascularization, promoting the formation of new blood vessels and enhancing nutrient delivery. PDRN leads to significant and rapid pain relief and improves structural integrity by enhancing collagen synthesis and mechanical strength. It also restores functional capacity by increasing joint range of motion and performance. Its efficacy has been demonstrated in various tendinopathies, including Achilles tendinopathy, plantar fasciitis, and rotator cuff disease. It has been shown to deliver superior outcomes, including increased tensile strength, long-term pain relief comparable to corticosteroids, and improved shoulder mobility.

Figure 2 summarizes the key points: “The effects of PDRN treatment or therapy in tendinopathies.”

## 4. The Effects of PDRN Treatment or Therapy in Bone Regeneration

### 4.1. The Mechanisms of PDRN in Bone Regeneration

PDRN promotes bone regeneration through angiogenesis, osteoblast stimulation, and modulation of local inflammation, all of which are important for effective bone healing and augmentation [13,14]. In an in vivo experimental study, PDRN enhanced early bone formation during lateral bone augmentation, especially when combined with collagenated biphasic calcium phosphate (CBCP) and a collagen membrane [13,14].

The proposed mechanisms of PDRN in bone regeneration include the following:

▪ Angiogenesis promotion: PDRN increases VEGF and other growth factors, thereby supporting new blood vessel formation and improving the delivery of oxygen, nutrients, and progenitor cells to the repair site [13,14].
▪ Osteoblast stimulation: PDRN is believed to stimulate osteoblast activity and promote their proliferation and differentiation, thereby enhancing new bone matrix synthesis [13,14,35].
▪ Anti-inflammatory effects: PDRN reduces inflammation and creates a more favorable environment for bone repair by activating A2ARs and inhibiting pro-inflammatory signaling pathways, such as NF-κB and PDRN [13,14].
▪ Nucleotide provision: PDRN supplies purine and pyrimidine bases, deoxyribonucleosides, and deoxyribonucleotides that cells use via the salvage pathway to synthesize DNA and RNA, thereby supporting the proliferation and differentiation of damaged or ischemic tissues and promoting bone repair [7,12,13,15].

### 4.2. Preclinical Studies in Bone Regeneration

In a preclinical beagle dog study, extraction of the third or fourth premolars was followed by the creation of 5 mm high dehiscence defects and immediate placement of 3D-printed implants [13,14]. The defects were grafted with either CBCP and a collagen membrane or CBCP soaked in PDRN and a collagen membrane, and samples were collected at defined time points to assess early bone formation [13,14]. This study evaluated the bone volume, total volume, new bone area, and residual bone substitute material, and the overall findings showed that PDRN facilitated early bone formation [13,14].

Overall, PDRN appears to be a promising adjunct for bone augmentation procedures, with potential applications in orthopedic and dental bone repair [13,14]. Further analysis of the original quantitative and histological data is still needed to fully define the effect size and clinical relevance [13,14].

In addition, PDRN accelerates bone tissue repair and growth. In vitro studies have shown that PDRN at 100 µg/mL promotes the proliferation of cultured human osteoblasts and increases alkaline phosphatase activity, effects that are suppressed by the A2AR antagonist DMPX [2,11,36]. In calvarial defect models, PDRN combined with human demineralized dentin matrix enhances osteoinduction, with histological evidence of new bone matrix deposition and calcification, increased numbers of bone-forming cells and blood vessels, and expansion of new bone growth on dentin particles [18,37]. PDRN has also shown anti-osteonecrotic effects by reducing necrotic bone formation, improving vascular density and bone remodeling, and suppressing the expression of IL-1β, MMP-3, and MMP-7 through ERK inhibition [18]. In addition, increased expression of CD31, transglutaminase-II, and angiopoietin suggests that improved angiogenesis is a key contributor to PDRN’s osteogenic effects [9]. In inflammatory periodontitis models, PDRN reduces tissue damage, lowers cytokine levels, decreases the expression of apoptotic proteins, and preserves alveolar bone quality [2]. Studies using ceramic scaffolds have also reported enhanced bone formation with PDRN, particularly when combined with bone morphogenetic proteins [11,38].

**Figure 3:**
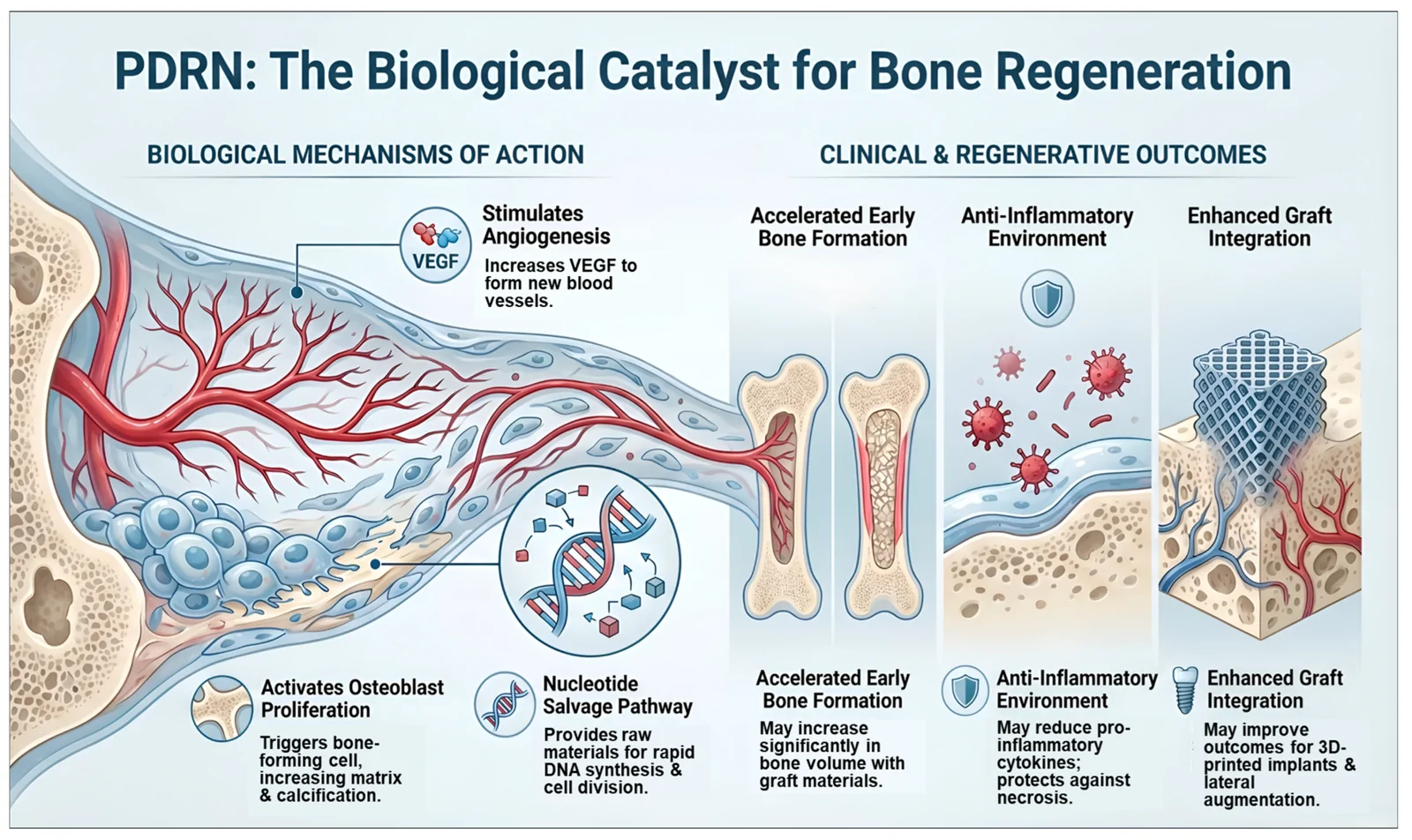
PDRN acts as a biological catalyst for bone regeneration via several mechanisms. PDRN stimulates angiogenesis by increasing VEGF to promote the formation of new blood vessels. PDRN also activates osteoblast proliferation, thereby triggering the synthesis of bone-forming collagen and matrix calcification. Furthermore, it provides the necessary raw materials via the nucleotide salvage pathway to accelerate DNA synthesis and cell division. PDRN may accelerate new bone formation, reduce pro-inflammatory cytokines to create an anti-inflammatory environment, and enhance graft integration, thereby improving outcomes of 3D-printed implants and bone augmentation procedures.

Figure 3 summarizes the key points: “The effects of PDRN treatment or therapy in bone regeneration.”

## 5. Preliminary and Exploratory Applications in Ligament Injuries

### 5.1. The Mechanisms of PDRN in Ligament Injuries

PDRN treatment shows therapeutic potential for ligament injuries by promoting tissue regeneration, modulating inflammation, and supporting functional recovery, largely through A2AR activation and nucleotide provision via the salvage pathway [12]. Although the cited literature focuses primarily on tendon disorders, these mechanisms are also relevant to ligament healing because tendons and ligaments share similar connective tissue composition and injury responses [12,25].

Specifically, PDRN may support ligament healing through several mechanisms:

▪ Inflammation modulation: Ligament injury is typically followed by an acute inflammatory phase that can impair healing if prolonged or excessive. PDRN acts as an adenosine A2AR agonist and inhibits pro-inflammatory signaling pathways, including NF-κB [12]. This reduces the production of inflammatory mediators and helps to create a more favorable environment for tissue repair [7,12,23]. By limiting chronic inflammation, PDRN may reduce the risk of fibrotic healing and impaired ligament function.
▪ Tissue regeneration and repair: In injured or ischemic tissues, where de novo nucleotide synthesis may be compromised, PDRN provides purine and pyrimidine bases, deoxyribonucleosides, and deoxyribonucleotides that sustain salvage pathway-dependent nucleic acid synthesis [7,12,15]. By promoting cell proliferation and differentiation, PDRN may help reconstruct damaged ligament fibers and improve their structural integrity [7,12].
▪ Promotion of angiogenesis: An adequate blood supply is essential for healing dense connective tissues such as ligaments. PDRN promotes angiogenesis by stimulating the production of VEGF and other growth factors [12]. Improved vascularization enhances oxygen and nutrient delivery and supports waste removal, all of which are necessary for effective tissue repair [12].
▪ ECM remodeling: PDRN promotes collagen synthesis, a key component of ligament repair and remodeling [12]. As collagen is the main structural protein in ligaments, proper synthesis and organization are essential for restoring mechanical strength and tissue integrity. By modulating ECM remodeling, PDRN may favor organized ligament repair over scar formation, resulting in inferior mechanical properties [12].

PDRN may improve the tendon–bone interface and enhance ligament–bone healing, thereby reducing graft failure after reconstruction [1,24,32].

### 5.2. Preclinical and Clinical Studies in Ligament Injuries

In a preclinical case report, a 43-year-old woman with a near-complete ACL tear at the femoral attachment and a partial lateral collateral ligament tear; after five ultrasound-guided PDRN injections given at approximately 2-week intervals, she achieved complete pain relief and full range of motion at 3 months, with sustained improvement at 2 years and 5 months [39]. Although this is a single case and cannot be generalized, it suggests that PDRN may be a non-surgical regenerative option for ligament injuries and is consistent with its known effects on collagen production and fibroblast differentiation [40]. A meta-analysis also showed that PDRN injections significantly reduced pain in patients with combined tendon or ligament disorders, providing broader support for their analgesic and regenerative potential in musculoskeletal soft tissue diseases [25].

Clinical studies have reported the beneficial effects of PDRN in patients with tendon or ligament pain, as summarized in meta-analyses [12,25]. Although these studies often combined tendon and ligament disorders, they suggested a shared therapeutic benefit across soft tissue injuries. However, detailed long-term histological and functional data specific to the different ligament injuries are limited.

Overall, the effects of PDRN on inflammation, cellular repair, and tissue remodeling are highly relevant to ligament pathology. Available evidence, particularly from meta-analyses of combined tendon or ligament pain, supports a favorable role for PDRN in these conditions and suggests that it may be a promising treatment option [12,25]. Further targeted preclinical and clinical studies are needed to clarify its long-term effects on functional recovery and histological healing of specific ligament injuries.

**Figure 4:**
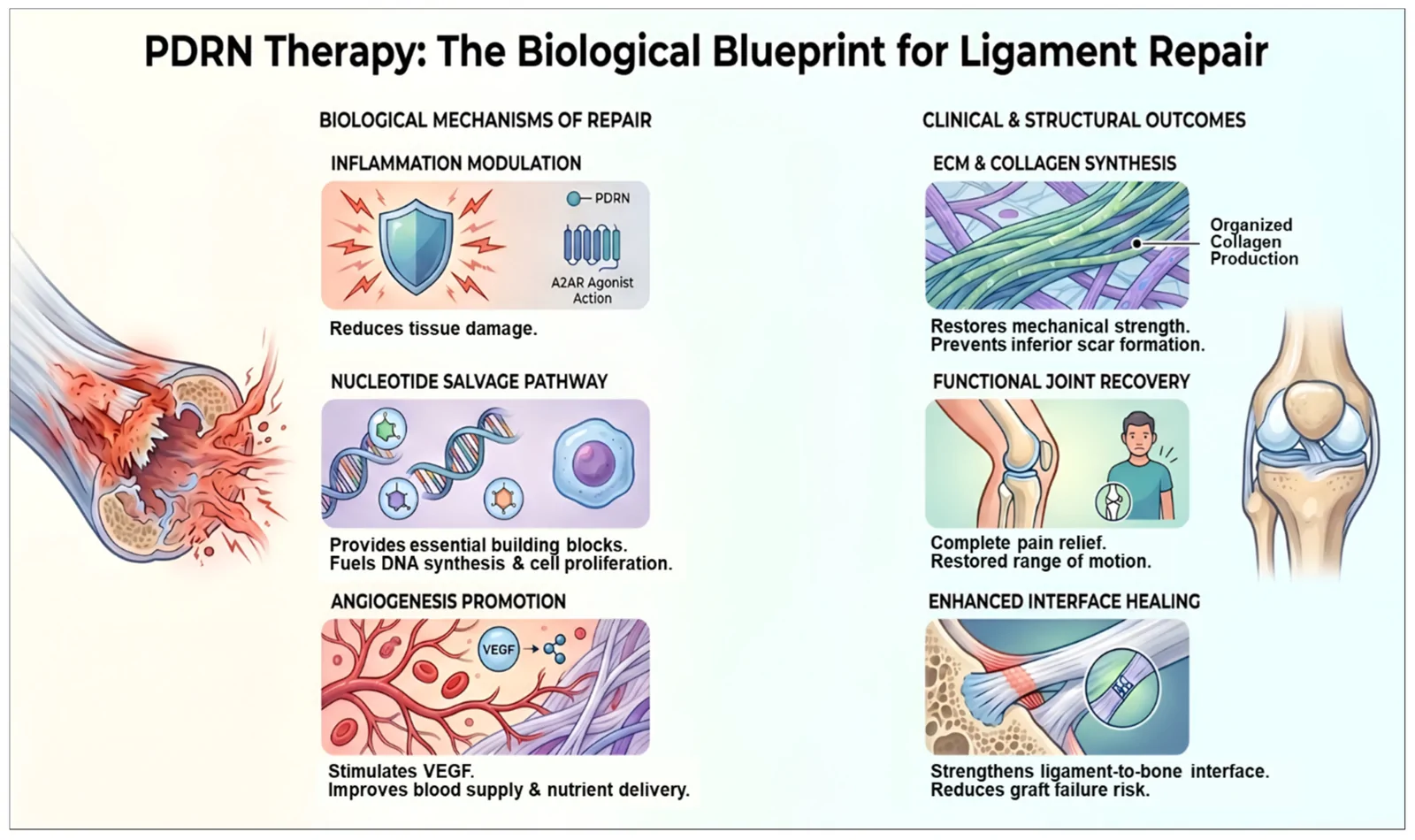
PDRN promotes ligament repair via various biological mechanisms. PDRN modulates inflammation by activating A2ARs and reducing tissue damage through its anti-inflammatory effects. PDRN provides essential nucleotides via the nucleotide salvage pathway, thereby fueling DNA synthesis and cell proliferation. It also stimulates VEGF-mediated angiogenesis, thereby improving blood supply and nutrient delivery. PDRN enhances ECM and collagen synthesis to restore mechanical strength and prevent poor scar formation. These processes result in complete pain relief and functional joint recovery, restoring range of motion. Furthermore, PDRN strengthens the ligament-to-bone interface, thereby reducing the risk of graft failure.

Figure 4 summarizes the key points: “The effects of PDRN treatment or therapy in ligament injuries.”

## 6. Theoretical Rationale and Emerging Findings in Spinal and Radicular Disorders

### 6.1. The Mechanisms of PDRN in Spinal and Radicular Disorders

Specific data on the effects of PDRN in spinal and radicular disorders are unavailable. The current literature predominantly describes the use of PDRN in tendon disorders, osteoarthritis, and bone regeneration, as well as its general anti-inflammatory and tissue-repairing properties in musculoskeletal tissues [7,12,13,14,15,17,23,24,25,41]. Although PDRN mechanisms, such as A2AR activation and nucleotide supply via the salvage pathway, support regenerative and anti-inflammatory effects across multiple tissues, their direct therapeutic impact on spinal and radicular disorders has not been explicitly addressed in the cited references [12,15].

PDRN’s established mechanisms include suppressing inflammation by inhibiting pro-inflammatory pathways such as NF-κB and supporting tissue repair by providing DNA and RNA precursors [15]. It also stimulates angiogenesis by increasing VEGF production and contributes to ECM remodeling by promoting collagen synthesis [24]. These effects have been observed in rat Achilles tendon injury models, in which PDRN reduces inflammation and apoptosis [23], and in rotator cuff repair models, in which PDRN improves tendon healing and attenuates fatty degeneration [24,41]. PDRN has also shown the potential to reduce cartilage degradation and support chondrogenic differentiation in hBMSCs, suggesting its utility in osteoarthritis [15]. Its role in early bone formation has been demonstrated using in vivo experimental models of lateral bone augmentation [13].

Given its broad regenerative and anti-inflammatory properties, PDRN could theoretically benefit patients with spinal and radicular disorders, which commonly involve inflammation, nerve compression, and tissue damage. For example, its anti-inflammatory effects might ameliorate nerve-root inflammation in radiculopathy, and its tissue-repairing activity could support the healing of injured spinal structures. However, these inferences are drawn from general mechanisms rather than direct findings from the cited literature on spinal or radicular diseases.

### 6.2. Preclinical and Clinical Studies in Spinal and Radicular Disorders

Dedicated preclinical and clinical studies targeting these conditions are required to establish the effects of PDRN in spinal and radicular disorders. Such studies should evaluate the effects of PDRN on neuroinflammation, nerve regeneration, intervertebral disc health, and pain management in the spinal environment. The existing literature, while underscoring PDRN’s promise in other orthopedic indications, does not provide sufficient data to assess its efficacy or underlying mechanisms, specifically in spinal and radicular disorders. Consequently, visual materials or quantitative outcomes for PDRN under these conditions cannot be derived from the available sources. Visual materials require reference to the original research literature.

Transforaminal epidural glucocorticoid injection is a commonly used intervention for lumbosacral radiculopathy; however, it may exacerbate the symptoms in patients with type 2 diabetes. PDRN has been proposed as a potential non-steroidal alternative in this setting. The first preliminary case report of fluoroscopically guided transforaminal epidural PDRN injection involved a 44-year-old male diabetic patient with left L4–5 foraminal disc protrusion and L5–S1 subarticular disc protrusion who showed good clinical improvement over 6 months without complications [42]. This case illustrates PDRN’s potential as a safe alternative for patients in whom corticosteroids are contraindicated, including those with poorly controlled diabetes [40]. Its anti-inflammatory and neuroprotective effects, mediated by A2AR activation and cAMP-PKA-CREB signaling, provide a biologically plausible rationale for use in neuromusculoskeletal pain conditions, although controlled trials are still needed to confirm its efficacy in spinal disorders [18].

Activation of A2AR by PDRN improves neural microvascular blood flow and supports spinal cord protection after injury [11,43]. Transforaminal epidural PDRN injections have been used effectively to manage lumbosacral radiculopathy when corticosteroids are not suitable [42]. Meta-analyses indicate that while epidural steroid injections mainly provide short-term relief, PDRN may contribute to sustained neural recovery [44].

**Figure 5:**
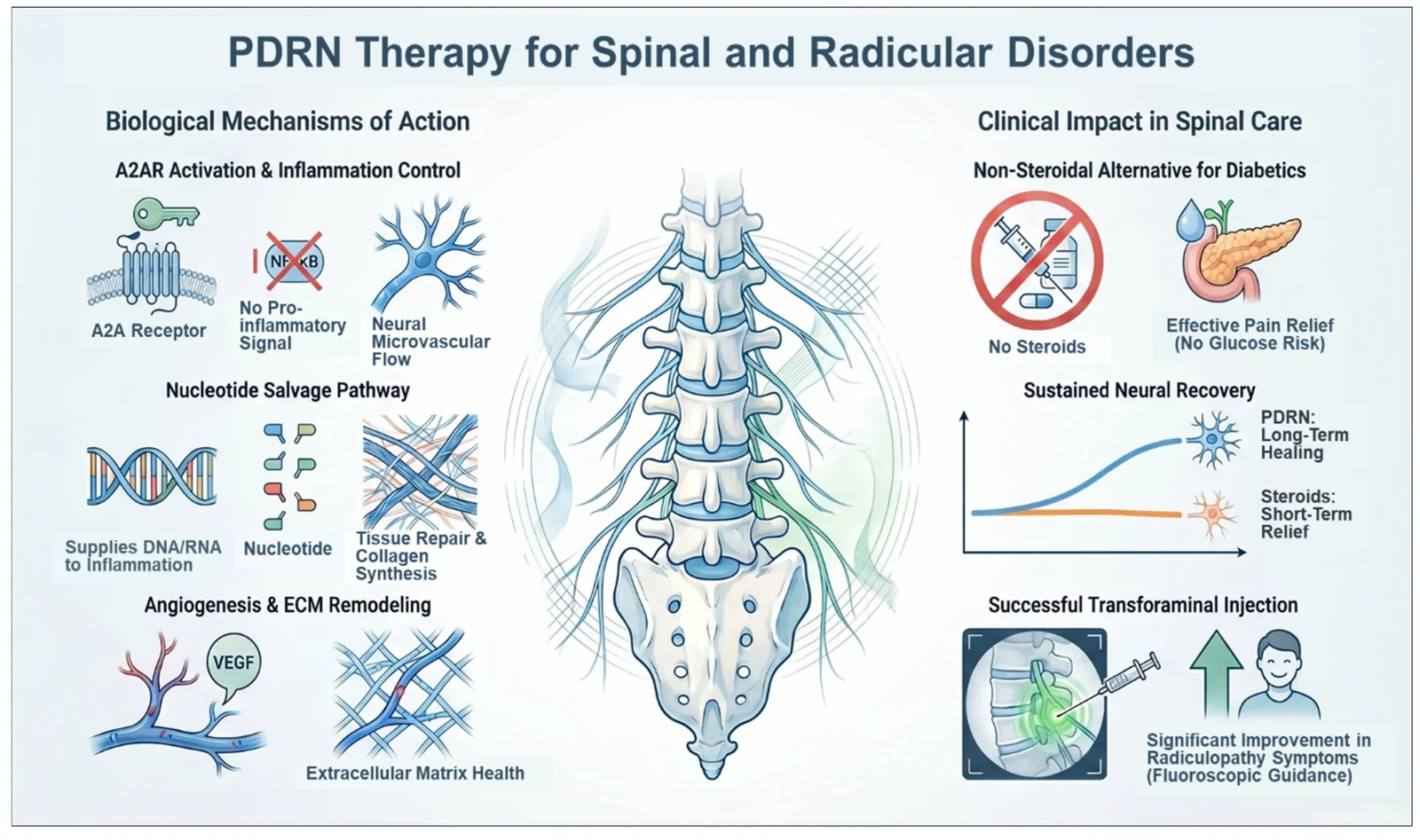
Theoretical model and preliminary biological rationale of PDRN for spinal and radicular disorders. Activation of A2ARs inhibits pro-inflammatory signaling, thereby reducing neural inflammation and promoting microvascular flow. Through the nucleotide salvage pathway, PDRN provides the essential DNA and RNA precursors for tissue repair and collagen synthesis. PDRN also stimulates VEGF-mediated angiogenesis, thereby improving ECM health and supporting neural recovery. As a non-steroidal alternative, PDRN provides effective pain relief for diabetic patients without posing risks to glucose metabolism. Successful transforaminal injection under fluoroscopic guidance significantly improves radiculopathy symptoms and promotes long-term neural healing, providing greater relief than steroids offer in the short term. Sustained recovery relative to steroids represents a hypothesis requiring validation in prospective randomized trials.

Figure 5 summarizes the key points: “The effects of PDRN treatment or therapy in spinal and radicular disorders.”

## 7. Safety Profile and Tolerability

Comprehensive preclinical and clinical evidence supports PDRN’s safety profile and tolerability. Acute and chronic toxicity studies in mice and rats showed no toxic effects in the brain, liver, lungs, skeletal muscle, or heart and no treatment-related mortality at 8 mg/kg [2]. In clinical trials with diverse indications, PDRN was associated with safety and tolerability, with no reported systemic side effects, anaphylaxis, or significant local reactions [7]. A 5-year post-marketing surveillance study involving more than 300,000 PDRN-dispensed prescriptions further confirmed an outstanding safety profile at the population level [2,7].

As a non-steroidal agent, PDRN is particularly advantageous in diabetic patients who cannot receive corticosteroid injections and in populations at risk of tendon rupture and tissue degeneration with repeated corticosteroid use [7]. However, PDRN is not recommended during pregnancy owing to the lack of supporting clinical data and is contraindicated in individuals with known hypersensitivity to PDRN, although no allergic reactions have been reported in the literature [9].

## 8. Limitations and Future Directions

Despite growing evidence for PDRN’s clinical utility in orthopedics, several limitations remain in the current literature. Most clinical studies are retrospective observational studies, case reports, or small-scale trials, with relatively few large-scale, multicenter randomized controlled trials, thereby limiting causal inference [40]. A notable methodological concern is that all 18 studies included in their review reported positive outcomes with no negative effects, raising the possibility of publication bias and highlighting the need for rigorous designs that systematically capture potential harms [40]. Moreover, there is substantial heterogeneity in PDRN dosage, injection frequency, and routes of administration. Although PDRN is typically administered three to five times, the optimal dose for musculoskeletal conditions remains unstandardized, unlike in dermatologic applications, where dosing is better defined [1].

Future research should include multicenter randomized controlled trials with standardized dosing protocols, longer follow-ups, and larger sample sizes to more robustly evaluate PDRN’s efficacy in specific musculoskeletal disorders [12]. The synergistic potential of combining PDRN with other modalities, such as ESWT, PEMF, hyaluronic acid, mesenchymal stem cells, and microcurrent therapy, deserves systematic assessment using well-designed clinical protocols, especially in light of promising preclinical data [7]. In addition, the development of PDRN-incorporated scaffolds based on bioceramics, hydrogels, and cell- or tissue-derived materials represents an emerging frontier in tissue engineering that could expand PDRN’s role in bone and cartilage regeneration [45].

Table 2 explicitly categorizes each musculoskeletal indication by its highest level of available evidence and current translational maturity, ensuring that exploratory indications (such as spine and ligament applications) are clearly distinguished from established ones (such as knee OA and tendinopathies).

### Comparative Biologics Context and Translational Barriers

When evaluating PDRN alongside established biotherapies, such as HA, platelet-rich plasma (PRP), and MSCs, several operational and biological differences emerge (Table 3). PDRN offers an advantage as a standardized, off-the-shelf pharmaceutical agent, avoiding the donor-to-donor variability inherent to autologous PRP or the high cost and regulatory complexity of MSCs. However, clinical translation remains hampered by several key barriers:

▪ Dosing and Protocol Heterogeneity: Unlike HA, which follows standardized intra-articular administration guidelines, PDRN regimens vary widely in frequency (1–5 injections) and concentration across trials.
▪ Patient Selection: Current evidence suggests optimal responsiveness in mild-to-moderate degenerative conditions (e.g., early-stage OA) or subacute tendinopathies, particularly in patients with metabolic comorbidities (such as diabetes) where corticosteroid use is restricted.
▪ Long-Term Efficacy: Existing literature rarely tracks outcomes beyond 6 to 12 months, highlighting the need for long-term registry studies.

**Table 3:** Comparative overview of common orthopedic biotherapies.

Biologic Modality	Primary Mechanism	Product Standardization	Primary Limitation
PDRN	A2AR agonism + Nucleotide salvage pathway	High (Off-the-shelf drug)	Unstandardized orthopedic dosing protocols
HA	Viscosupplementation & joint lubrication	High (Off-the-shelf)	Predominantly symptomatic; limited structural repair
PRP	Autologous growth factor release	Low (High inter-patient variability)	Inconsistent preparation and concentration protocols
MSCs	Paracrine signaling & multi-lineage potential	Variable	High cost, complex regulation, donor age dependence

## 9. Conclusions

**Figure 6:**
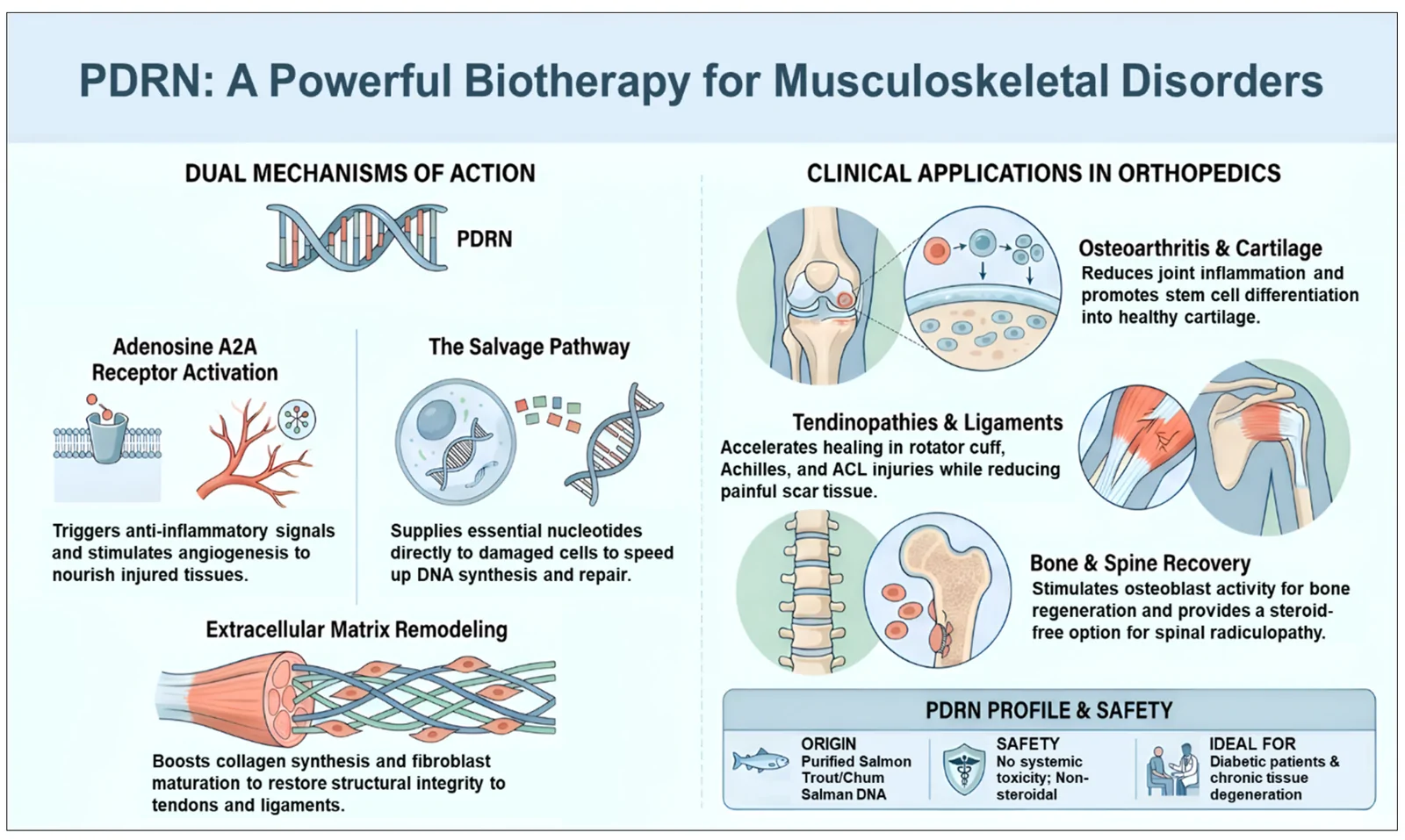
PDRN exerts therapeutic effects on musculoskeletal disorders through two mechanisms. Firstly, it activates A2ARs, which trigger anti-inflammatory signals and promote angiogenesis to nourish injured tissues. Secondly, PDRN provides vital nucleotides via the salvage pathway, accelerating DNA synthesis and tissue repair. Furthermore, PDRN enhances ECM remodeling by boosting collagen synthesis and fibroblast maturation, thereby restoring the structural integrity of tendons and ligaments. PDRN is effective in treating osteoarthritis and cartilage damage, reducing inflammation and promoting stem cell differentiation. It accelerates the healing of tendinopathies, such as those affecting the rotator cuff and Achilles tendon, and reduces the formation of painful scar tissue. Additionally, PDRN stimulates osteoblast activity to promote bone and spinal regeneration, providing an alternative to steroids for treating spinal radiculopathy.

PDRN is a promising, multifaceted regenerative agent whose well-characterized mechanism centers on A2AR activation and the salvage pathway, conferring anti-inflammatory, anti-apoptotic, pro-angiogenic, and tissue-repairing effects that are highly relevant to orthopedic practice [2]. Current evidence supports its clinical utility in a broad range of musculoskeletal disorders, including knee osteoarthritis, tendinopathies of the rotator cuff, Achilles tendon, plantar fascia, and common extensor tendon, as well as bone regeneration, ligament injuries, and spinal radicular pain, with consistent reports of pain reduction, functional improvement, and an excellent safety profile (Figure 6) [1,7]. The transition of PDRN from a primary dermatological agent to mainstream orthopedic therapy is supported by accumulating preclinical mechanistic data and clinical outcomes, although high-quality randomized trials remain essential to standardize protocols and define their optimal role within integrated musculoskeletal rehabilitation programs [12,45].

## Figures and Tables

**Table 1:** A comparison of the key characteristics between PDRN and HA.

Feature	PDRN	HA
Primary Mechanism	A2AR activation → anti-inflammatory + angiogenesis + tissue repair	Viscoelastic supplementation → lubrication & shock absorption
Biological Target	Wound/joint microenvironment modulation	Synovial fluid biomechanics
Regenerative Potential	Moderate–high (indirect regeneration support)	Low (mainly symptomatic)
Onset of Effect	Gradual (weeks)	Moderate (weeks)
Duration of Benefit	Months	Months (variable)
Effect on Angiogenesis	Physiologic stimulation	Minimal
Effect on Inflammation	Immune reprogramming (resolution phase)	Minimal
Effect on Cartilage/Tendon Biology	Supports ECM synthesis & fibroblast activity	Protects the surface mechanically
Disease-Modifying Potential	Possible	Limited
Pain Relief Strength	Moderate	Moderate
Reproducibility	High (standardized product)	High
Safety Profile	Excellent	Excellent
Tumorigenic Risk	None	None
Cost	Moderate	Moderate
Regulatory Complexity	Low	Low
Typical Indications	Tendinopathy, osteoarthritis, chronic soft-tissue injury	Knee osteoarthritis, joint pain

**Table 2:** Summary of evidence maturity across musculoskeletal indications.

Indication	Highest Level of Evidence Available	Dominant Study Types	Clinical Maturity Status
Knee Osteoarthritis	Level I	RCTs, Systematic Reviews/Meta-analyses	Established/High
Tendinopathies	Level I–II	RCTs, Comparative Cohort Studies	Moderate–High
Bone Regeneration	Level IV–V	In vivo animal models, in vitro cell studies	Preclinical/Emerging
Ligament Injuries	Level IV–V	Isolated Case Reports	Exploratory/Low
Spinal Disorders	Level IV–V	Isolated Case Reports, Preclinical SCI models	Exploratory/Low

## Data Availability

No new data were created or analyzed in this study. Data sharing is not applicable to this article.
